# A Novel MOS Nanowire Gas Sensor Device (S3) and GC-MS-Based Approach for the Characterization of Grated Parmigiano Reggiano Cheese

**DOI:** 10.3390/bios6040060

**Published:** 2016-12-16

**Authors:** Veronica Sberveglieri, Manohar Prasad Bhandari, Estefanía Núñez Carmona, Giulia Betto, Giorgio Sberveglieri

**Affiliations:** 1 Sensor Lab, CNR, National Institute of Optics (INO), Via Valotti 9, 25133 Brescia, Italy; e.nunezcarmona@unibs.it (E.N.C.); giorgio.sberveglieri@unibs.it (G.S.); 2 Department of Information Engineering, University of Brescia, Via Branze 38, 25123 Brescia, Italy; m.bhandari@studenti.unibs.it (M.P.B.); giulia.betto@unibs.it (G.B.)

**Keywords:** nanowire gas sensor array, electronic nose, S3, SPME-GC-MS, Parmigiano Reggiano, cheese quality

## Abstract

To determine the originality of a typical Italian Parmigiano Reggiano cheese, it is crucial to define and characterize its quality, ripening period, and geographical origin. Different analytical techniques have been applied aimed at studying the organoleptic and characteristic volatile organic compounds (VOCs) profile of this cheese. However, most of the classical methods are time consuming and costly. The aim of this work was to illustrate a new simple, portable, fast, reliable, non-destructive, and economic sensor device S3 based on an array of six metal oxide semiconductor nanowire gas sensors to assess and discriminate the quality ranking of grated Parmigiano Reggiano cheese samples and to identify the VOC biomarkers using a headspace SPME-GC-MS. The device could clearly differentiate cheese samples varying in quality and ripening time when the results were analyzed by multivariate statistical analysis involving principal component analysis (PCA). Similarly, the volatile constituents of Parmigiano Reggiano identified were consistent with the compounds intimated in the literature. The obtained results show the applicability of an S3 device combined with SPME-GC-MS and sensory evaluation for a fast and high-sensitivity analysis of VOCs in Parmigiano Reggiano cheese and for the quality control of this class of cheese.

## 1. Introduction

The Parmigiano Reggiano cheese is the most renowned Italian cheese worldwide, acclaimed for its unique production, nutritional and organoleptic properties. It bears a Protected Designation of Origin (PDO) certification adhering to the EC Regulation 2081/92 [1], thus its production is governed by a strict standard following a traditional artisan procedure that takes place exclusively in the provinces of Parma, Reggio Emilia, Modena, Mantova, and Bologna in northern Italy [2]. This semi fat, additive-free, hard cooked cheese produced from raw and semi-skimmed cow’s milk must ripen for at least 12 months and up to 36 months [3,4]. The cattle producing the milk consume locally grown forage and the supply of silage and fermented feeds is not permitted. The manufacturing of this cheese remains unchanged for centuries and it represents an essential product in the agricultural economy of Italy. Parmigiano Reggiano Cheese Consortium (CFPR) controls the overall production of Parmigiano Reggiano starting from the grazing of the cow to the cheese manufacturing and ripening processes [5].

Flavor and aroma of the cheese are decisive factors for the consumer’s acceptance of the product. The enzymatic and biochemical activities (lipolysis, proteolysis, and glycolysis) that occur during cheese processing and ripening result in the formation of a wide range of volatile organic compounds (VOCs), differing in polarity and reactivity, contributing to the characteristic cheese flavor and texture [6,7,8]. The volatile aroma compounds of Parmigiano Reggiano cheese have been extensively studied and a large number of volatiles have been detected hitherto using various analytical techniques, as reported by different authors [9,10,11,12,13,14]. They include several classes of compounds: fatty acids, esters, aldehydes, ketones, alcohols, lactones, hydrocarbons, amines, pyrazines, and sulphur compounds [15].

Consequently, these compounds define the cheese quality, safety, ripening time, and sensory profile, serving sometimes as a signal of processing error as well [16]. From the published works, it is evident that the cheese flavor is not defined by a few character impact compounds, but is the result of the chemical interaction between a complex group of components. The authentication and characterization of high-commercial-value PDO cheeses such as Parmigiano Reggiano is needed because its quality continues to pose a challenge for cheese manufacturers.

In recent years, grated Parmigiano Reggiano cheese has been battling frequent adulteration and mislabeling. It is one of the most counterfeited foodstuffs in the world. Many of the imitators or faux Parmigiano Reggiano are produced in different countries—with generic names such as Parmesan, Parmigiana, Parmesana, Parmabon, Real Parma, Parmezano, and Permesansan— and they are not subject to production regulations (www.parmigianoreggiano.com). Since PDO is not usually recognized outside Europe, Parmigiano Reggiano is generally known as Parmesan and all imitation products are termed as such and processed quite differently [17]. To this end, the consortium is beginning to use a transparent system to optimize the production, detect fraud, and protect the venerable brand.

Electronic nose, machine olfaction, or artificial olfaction technology has successfully been applied in many fields of food quality control and assessment, especially in flavor monitoring and classification of various cheeses with respect to their variety, geographical origin, and ripening stage [18,19,20,21,22,23,24,25]. It consists of an array of gas sensors with specificity and an automated pattern recognition system to recognize simple or complex odors [26,27].

The electronic nose signals are analyzed by means of graphical or multivariate analysis depending on the available data and the type of results obtained [28]. Most of the classical instrumental techniques and chemical methods used are not only expensive and complex but also time consuming. However, from the commercial point of view, a sensor device system should be simple, low cost, and rapid with self-operating artificial intelligence meant for an easy and efficient screening method [29]. There is a growing need for such an alternative device to selectively measure and evaluate the quality parameters of grated Parmigiano Reggiano cheese.

Solid phase microextraction-gas chromatography-mass spectrometry (SPME-GC-MS) yields information about the identification and concentration of volatile compounds present in a cheese sample without pre-treatment, also about the relationship between different chemical classes of the volatiles and how they present the perceived sensory attributes. The identification of unique markers help in resolving the authenticity issues of the PDO cheeses [30]. Moreover, SPME-GC-MS coupled with an electronic nose analysis can provide a better understanding of the distribution of cheese volatile compounds into classes and track the cheese quality [31].

In the present study, a novel nanowire gas sensor device Small Sensors System (S3) (SENSOR Laboratory, Brescia, Italy) housing six metal oxide semiconductor gas sensors was used to evaluate and characterize the volatile organic compounds and the quality of Italian Parmigiano Reggiano cheese. The S3 system was applied directly on grated cheese samples at different ripening times. The correlation between S3 and SPME-GC-MS analysis helped to identify the specific volatile markers of this type of cheese that could be useful to distinguish Parmigiano Reggiano from other cheese varieties and to monitor its adulteration.

## 2. Materials and Methods

### 2.1. Sampling

A total of 25 grated Parmigiano Reggiano cheese samples of certified origin, both flatland and mountain, and with different ripening times were provided by Consorzio del Formaggio Parmigiano Reggiano (CFPR), Reggio Emilia, Italy (Table 1). The samples were of undegraded and degraded organoleptic qualities as classified previously by an official panel of judges. The consortium guaranteed the categories and the geographical origins of the cheese samples. The samples were stored at 4 °C prior to analysis. The organoleptic panel test and triangular test were performed on one batch of samples.

The pH of the stored cheese samples was checked with a pH meter pH 7 (XS Instruments, Modena, Italy) directly on the cheese. The average pH of the cheese samples was 5.37, which remained almost constant over the ripening time. The electrode of the pH meter was pressed down on the cheese surface and speared into the cheese.

### 2.2. Sensory Analysis

All the 25 samples of Parmigiano Reggiano cheese were evaluated through the quantitative descriptive analysis and triangle tests by a sensory evaluation panel consisting of nine trained panelists. Sensory attributes of the grated cheese samples were determined by evaluating the visual (color) and olfactive (odor intensity, butter/milk, acidic, astringent, pungent, putrid, mold, propionic acid, and butyric acid) descriptors by profile method. The panelists were asked to specify a numerical score on a seven-point scale (1 = weak or no intensity, 7 = strong intensity) for the intensity of the descriptive properties [32]. The samples were presented at random and evaluated separately.

Triangle tests were performed to evaluate the differences in sensory characteristics of the cheese samples. The samples were coded in a uniform manner, using three digits: 1, 2, and 3. Each trial was composed of three different samples, each with a different code. Two of the samples were the same cheese and one was different. All the possible sequences of the three samples were given to the panelists and they were forced to indicate which sample out of the three was different from the other two.

### 2.3. S3 Instrumentation

**Figure 1:**
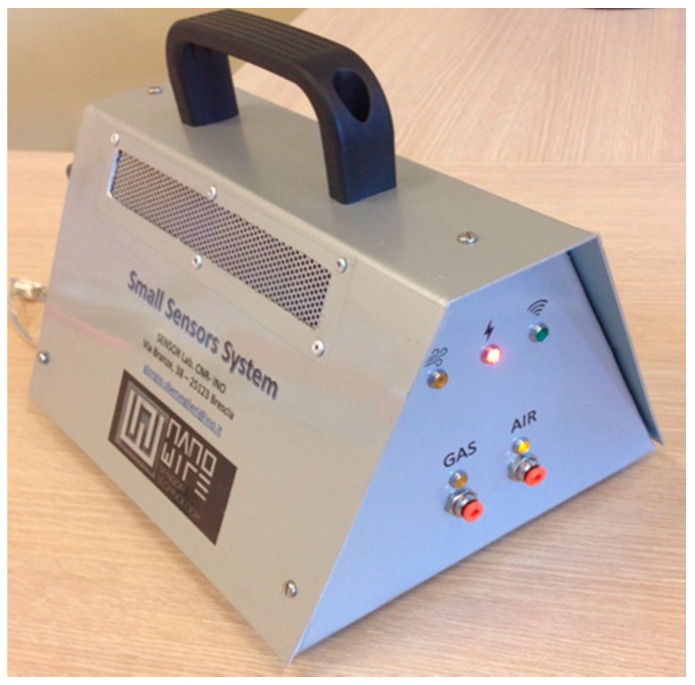
Portable S3 sensor device developed at SENSOR Laboratory, Brescia, Italy.

A new state-of-the-art nanowire gas sensor device Small Sensors System (S3) is presented for a quick and high-throughput quality and VOCs analysis of the Parmigiano Reggiano cheese samples considered in this work. The device has been completely designed and constructed at SENSOR Laboratory, Brescia, Italy. It is a miniaturized portable, compact, and automatic instrument. The system is designed in such a way that a greater amount of information can be gathered through a user-friendly control panel. It comprises an array of MOS gas sensors, flow sensors, temperature and humidity sensors, and actuators (valves, pumps), all embedded in a stainless steel cell. The sensing elements are Zinc and Tin oxide crystalline nanowires and thin films that have been fabricated and incorporated in the S3 sensor network. In particular, there are six metal oxide semiconductor gas sensors, three of them prepared with the nanowire technology [33] and the remaining three with the Rheotaxial Growth and Thermal Oxidation (RGTO) thin film technology [34]. Out of the three nanowires in the array, two of them are ZnO sensors with different operating temperatures and the third one is a SnO_2_ sensor. Regarding RGTO sensors, the first one is constructed based on a blend of SnO_2_ and MoO_3_, the second one is a SnO_2_ sensor catalyzed with Ag nanoparticles, and the third one consists of a blend of SnO_2_ and WO_3_. Each sensor is deposited on an Alumina substrate and doped with a Platinum heater on the sides. All the sensors in the array are unspecific. A photograph of the S3 device is shown in Figure 1, and a deep description of the sensors is explained in Table 2.

The excellent characteristics of metal oxide nanowires in terms of sensitivity, selectivity, and stability toward different molecules, 1-dimensional (1-D) nanostructure, low power dissipation, and content production costs allowed their use for the manufacture of S3 device as a versatile gas detection equipment [35]. The instrument was also connected with an autosampler headspace system HT280T (HTA srl, Brescia, Italy), bearing a 40-loading-site carousel and a shaking oven to equilibrate the sample headspace in order to provide a high amount of replicate for obtaining a consistent dataset. 

The operation principle of S3 is based on the analysis of the head space (HS) of a given sample. This volatile fraction is formed inside a vial during the achievement of the equilibrium between the solid phase and the vapor phase. The creation of the HS depends on the test substance (vapor pressure) and the conditioning temperature of the sample, which plays a decisive role determining the concentration of volatile compounds.

When compounds are extracted at the equilibrium point between the solid phase and the vapor phase the measurements are done in static HS, while if the extraction occurs continuously, it is called dynamic HS. In this case, the balance achieved between the solid phase and the vapor phase occurs during the measurement. The volatile fraction is then aspirated and transported to the sensor chamber where it will be analyzed.

**Figure 2:**
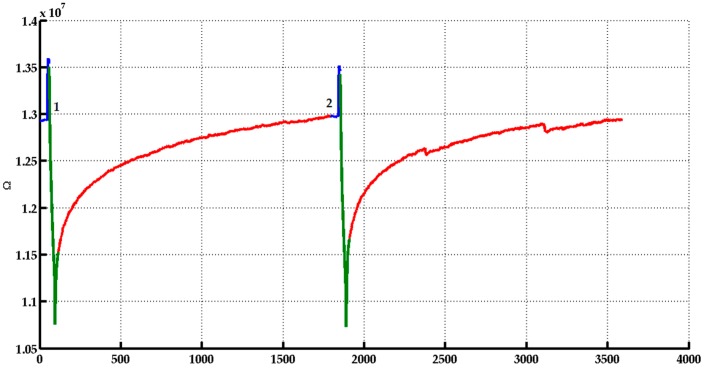
Graph showing the three steps of a measurement done with a MOX Nanowire SnO_3_ sensor, with before step in blue, during step in green, and after step in red. In the picture are represented two measurements of two cheese samples with 12 months of ripening and undegraded quality. The X-axis represents the time in seconds and the Y-axis the ohmic resistance.

- The analysis timeline can be divided into three different steps (Figure 2):
- Pre-injection/Before: At this step, a continuous flow of previously filtrated chromatographic air passes by the sensor chamber.
- Injection/During: The sample HS is flowed in the sensor chamber.
- Restoration/After: It starts when the injection period is finished, during this step filtered chromatography air is flowed into the sensor camber. In this time, the sensor restores the original condition of the base line.

These steps are preceded by a step of warm-up that allows the achievement of the base line for the entire system. The air is taken into the sensor chamber using a pump through a needle valve. That is used to adjust the total airflow, which is measured by a flowmeter downstream of the pump.

The chamber is also monitored with a thermostat to prevent the temperature of the surrounding environment from affecting the sensor responses. The volume of the chamber should be as small as possible. In this case, 20 mL to avoid the concentration gradient of the volatile fraction inside the chamber.

The chamber, and the connection between the element tires are made using steel pipes, in order to obtain a lower absorption of volatile substances that may then be released during subsequent measurements.

For S3 analysis, 2 g of grated cheese were taken in a 20 mL chromatographic vial for each sample, sealed with Silicon-PTFE septum, crimped with an aluminum crimp and then subjected to analysis. The equilibrium of the HS was reached thanks to the oven where the samples were shaken for 10 min at 40 °C.

To visualize the quantified complex data matrix acquired by the S3 in terms of resistivity changes and the relationships between different cheese samples and their volatile composition, quality, and ripening time, a multivariate statistical approach involving principal component analysis (PCA) was carried out. It was operated with a user-friendly Data Editor software and data were processed by EDA software developed in MATLAB at SENSOR laboratory. PCA is a linear combinatorial process whose core function is to reduce the complexity of the dataset by allowing the information to be shown in a narrow dimension. The result is derived from the variance of the dataset without any information on the classification of samples.

### 2.4. Analysis of Volatile Compounds by SPME-GC-MS

The volatile components of Parmigiano Reggiano cheese were extracted and identified by the headspace SPME-GC-MS method. A Shimadzu Gas Chromatograph GC2010 PLUS (Kyoto, KYT, Japan) interfaced with a Shimadzu single quadrupole Mass Spectrometer MS-QP2010 (Kyoto, KYT, Japan) ultra and a HT280T auto sampler was used to analyze the cheese headspace compounds.

All extractions were carried out using a DVB/carboxen/PDMS (divinylbenzene/carboxen/polydimethylsiloxane) stable flex (50/30 μm) (Supelco Co. Bellefonte, PA, USA) SPME triphasic fiber. The extracted volatile compounds of the cheese samples were separated by a low-polarity capillary column (DB-WAX, Agilent Technologies, Santa Clara, CA, USA). The conditioning of the sample was carried out maintaining the sample at 50 °C for 10 min in the HTA280T oven, the extraction of the compounds from the headspace was done by exposing the triphasic fiber for 15 min at the same temperature. The chromatogram was recorded with the following temperature program: 40 °C held for 8 min, linear gradient 4 °C/min up to 190 °C and held for 5 min, followed by a rise from 190 °C to 210 °C at 5 °C/min, for thermal desorption of the analytes. The carrier gas used was helium with a flow rate of 1 mL/min. The selection of the fiber type, extraction and operation conditions were the same as in this study [5]. The eluted compounds were identified by comparing them with the compounds listed in the National Institute of Standards and Technology (NIST) mass spectral database. Peak areas were calculated from the total ion current. For volatile compounds analysis, the samples of cheese (2 g) were weighed in 20 mL chromatographic vials sealed with Silicon-PTFE septum, crimped with an aluminum crimp, and then subjected to SPME-GC-MS experiment.

## 3. Results

### 3.1. Sensory Analysis

**Figure 3:**
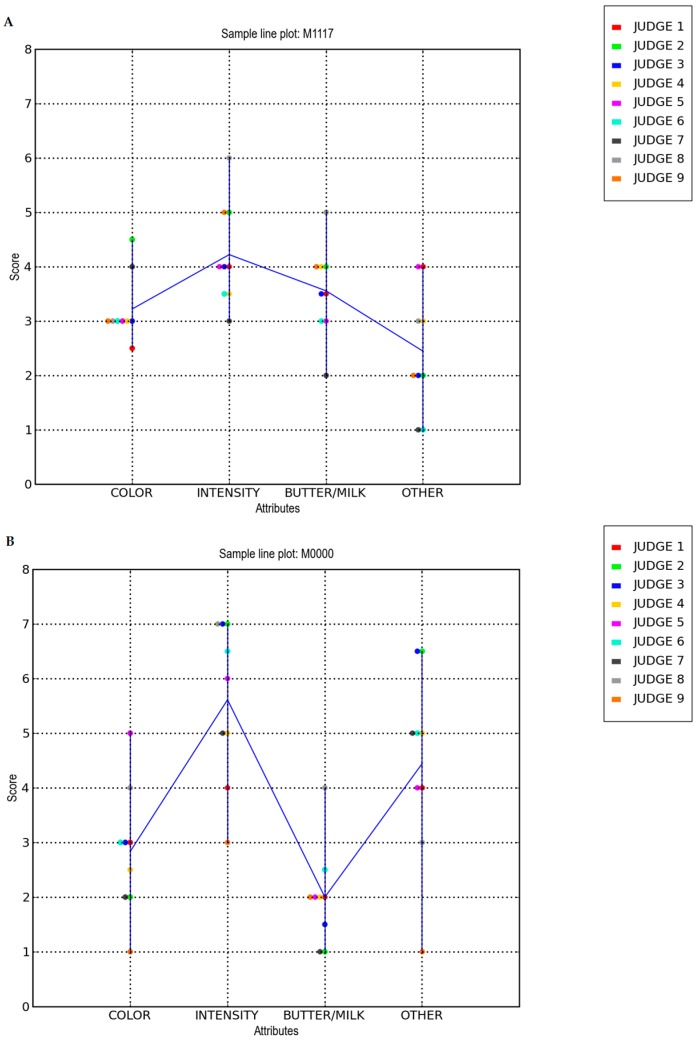
Sample line plots from the descriptive sensory analysis of (**A**) an undegraded cheese sample and (**B**) a degraded cheese sample. A score from 1 to 7 was given by a group of nine panelists based on their sensory perception of both the cheese samples.

Sensory panel tests and triangle tests were performed to evaluate the overall differences among the cheese samples. The odor intensity and other smell descriptors of the cheese samples were evaluated based on the given score (Figure 3). The undegraded sample (Figure 3A) and degraded one (Figure 3B) are shown respectively.

**Figure 4:**
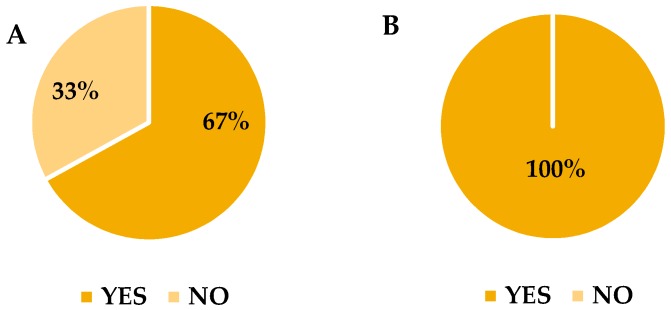
A pie chart showing the results of a triangle test between (**A**) an undegraded cheese sample and (**B**) a degraded cheese sample.

Figure 4 shows the results for a triangle test between the undegraded and the degraded cheese sample.

When the undegraded sample was different among the given three samples, only 67% of judges were able to detect the difference (Figure 4A) whereas when the degraded sample was different, all the judges were able to discriminate and identify the same (Figure 4B).

### 3.2. S3 (PCA) Analysis

**Figure 5:**
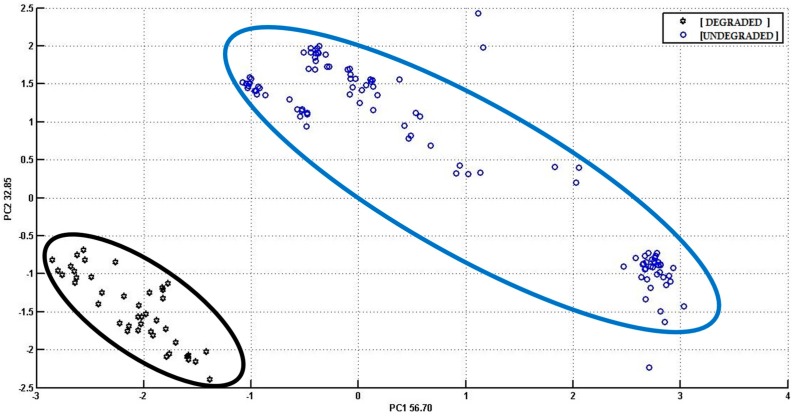
PCA score plot (PC1 versus PC2) from S3 measurements related to the sensor array response to the volatiles of degraded and undegraded cheese samples showing the clusters represented by black and blue circles, respectively. Explained variance: PC1 = 56.70%, PC2 = 32.85%.

Principal component analysis (PCA) was performed on the data acquired by S3 analysis. In the first PCA score plot (Figure 5), a clear discrimination between the earlier labeled undegraded and degraded cheese samples is shown.

**Figure 6:**
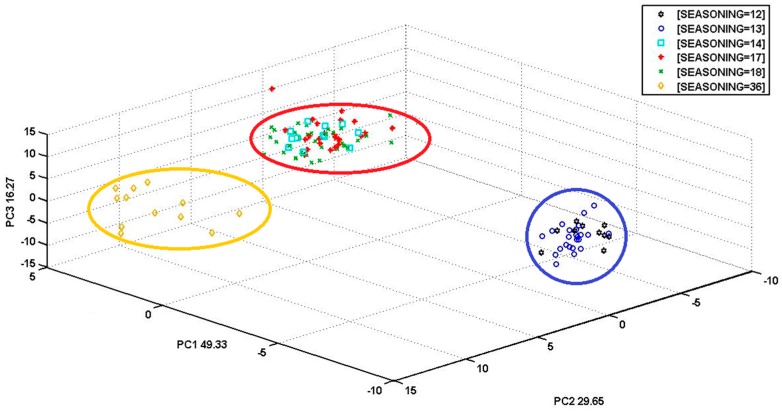
This is a PCA score plot from S3 measurements related to the sensor array response to the volatiles of cheese samples with different ripening times (in months), shown inside the clusters represented by red, blue, and yellow colors. The samples with 12 and 13 months of ripening are grouped within the blue cluster; the samples with 14, 17, and 18 months of ripening are grouped within the red cluster; and the samples with 36 months of ripening are grouped within the yellow cluster. Explained variance: PC1 = 49.33%, PC2 = 29.65%, PC3 = 16.27%.

In Figure 6, a PCA score plot for the ripening time of different cheese samples is reported. Also in this case, PCA carried out on the sensor array response showed convincing results concerning the distinction between the different cheese samples with different degrees of maturation (in months).

### 3.3. Volatile Compounds of the Parmigiano Reggiano Cheese (SPME-GC-MS Results)

The results of the headspace SPME-GC-MS measurements showed the presence of several volatile components in the Parmigiano Reggiano cheese contributing to the typical aroma of this cheese. About 150 key aroma compounds were identified in the cheese samples under study and the main compounds are listed in Table 3. The major categories of volatile compounds identified were aldehydes, ketones, free fatty acids, esters, hydrocarbons, alcohols, esters, lactones, aromatic compounds, amines, and pyrazines.

**Figure 7:**
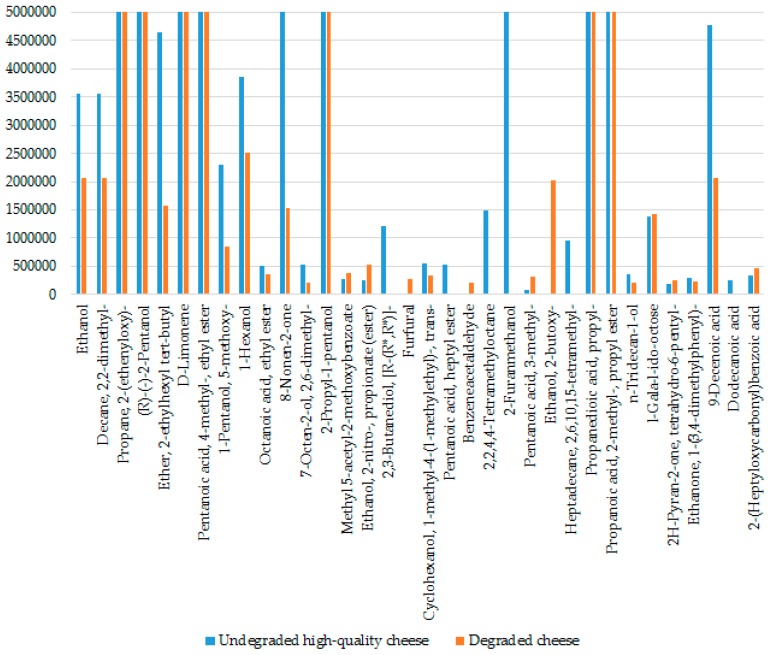
A histogram representing the VOCs from an undegraded high-quality cheese sample (blue) and a degraded Parmigiano Reggiano cheese sample (orange) identified by SPME-GC-MS analysis.

Figure 7 represents a histogram obtained from the SPME-GC-MS analysis showing the aroma compounds of representative undegraded and degraded Parmigiano Reggiano cheese samples.

The histogram depicted the major compounds from the Parmigiano Reggiano cheese as described above. There was a clear distinction in the flavor profiles between the undegraded and the degraded cheese samples.

## 4. Discussion

Regarding the sensory analysis, the overall odor intensity of the degraded sample was evaluated with higher values in comparison to the undegraded sample, a score less than 4 in the undegraded sample (Figure 3A) and a score more than 5 in the degraded one (Figure 3B). Similar results were obtained from the analysis of the degree of smell of butter/milk and other attributes such as acidic, pungent, putrid, mold, propionic acid, and butyric acid. In other words, the scores given by the panelists to the undegraded cheese sample were in strong contrast to the scores given to the degraded sample. The degraded sample B was aged for 36 months, hence a high odor intensity is expected (Figure 3B). In the same sample, the smell of butter/milk was evaluated with low values, nevertheless, with a high variability among the judges. This confirmed the actual and real differences between the two classes of cheese samples.

It was observed that the olfactory evaluation of cheese is a difficult task because it requires a lot of training and our perception of flavor and odor is extremely complex [36]. Despite some variation between the same class of samples, it was clearly noticed that the changes related to the quality and ripening period of Parmigiano Reggiano cheese took place in the odor and color of the product. These tests are generally used to make sure whether the samples are perceptively different. As such, they can segregate the sensory properties of the cheese hence providing important and useful information to the instrumental analyses, especially electronic nose and SPME-GC-MS technique.

On the subject of S3 analysis, the instrument, therefore, was able to distinguish the degraded cheese samples from the undegraded high-quality ones. The degraded samples are all grouped within the same cluster (black) on the left side of the graph, whereas the undegraded samples are grouped together within the same cluster (blue). This PCA explained 89.55% of the total explained variance within two PCs, which were represented by the cheese quality differences. A group of samples within the blue cluster were more scattered, probably accounting for variation between the aroma compounds, ripening time, and geographical origin of the cheese samples [23,37]. Few samples were outside the cluster which could be minimized by improving the sensitivity of the sensors [38]. In general, the clusters related to the quality of cheese samples are well separated, so that it is possible to detect the headspace volatile components based on the classes of cheese with the application of S3. The result is also in positive correlation with that of the triangular test.

From the graph in Figure 6, it can be easily concluded that the three clusters—blue, red, and yellow—diverge along particular directions according to different maturation period. In particular, the blue cluster groups the young cheese samples with 12 and 13 months of ripening; the red cluster includes the cheese samples with 16, 17, and 18 months of ripening; and the yellow cluster is represented by mature specimens cured for 36 months. The three clusters are very compact and well differentiated between them, therefore, the instrument was successful in sensing the differences between the aromatic profiles of the samples that constitute such groupings. This PCA model was more descriptive, the total explained variance was 95.25% within three PCs. The groups of samples at different ripening times were less scattered as well.

The reason for this difference in the aromatic profile between the samples is due to the fact that, during the aging process, the product undergoes organoleptic and biochemical changes reflected in the cheese aroma and flavor [39]. The distinctive sensory characteristics of Parmigiano Reggiano cheese can be considered as evolving throughout the ripening of the cheese. A typical Parmigiano Reggiano cheese with 12–15 months of ripening generally presents an aroma with a lactic note rather accentuated (milk, yogurt, butter), although not necessarily intense. This aroma is accompanied by the vegetable notes such as grass, boiled vegetables, and sometimes flowers or fruit, most probably from the cattle feeding. A 24–28 months ripened Parmigiano Reggiano also presents the aroma of milk with notes of melted butter, fresh fruits, citrus fruits, and meat broth. At 36–48 months, the cheese becomes very old, the aromatic notes of the spices (nutmeg, pepper) and dry fruits are highly dominant. The aroma of meat stock (stock cube) and lactic notes constitutes the flavor of Parmigiano Reggiano rind, an effect of long aging. The exploratory data analysis by PCA exhibited data reduction in the multivariate problem where variables were partially correlated [40].

In Table 3 the major categories of volatile compounds identified in the aromatic profile of Parmigiano Reggiano are represented. The main groups were aldehydes, ketones, free fatty acids, esters, hydrocarbons, alcohols, esters, lactones, aromatic compounds, amines, and pyrazines. Among the free fatty acids, the most abundant were propanoic, butanoic, hexanoic, octanoic, and decanoic acid. Other important compounds found were butanoic acid ethyl ester, hexanoic acid ethyl ester, octanoic acid ethyl ester, 2-heptanone, 2-nonanone, 3-methylbutanal, acetic acid, benzeneacetaldehyde, furfural, etc. The distinction in the volatile profiles of the cheese samples occurred during the ripening period. The relative amounts of some of the compounds or classes of compounds, mainly acids, increased during the cheese ripening. It should be noted that the ripening-induced changes in the cheese volatile constituents are, most probably, the result of the biochemical (lipolysis, proteolysis, glycolysis) [41] and microbiological transformation of native raw milk components and evaporation loss of the most volatile compounds. Therefore, the volatile Parmigiano Reggiano cheese profile is a result of a complex and dynamic equilibrium.

These components impart a characteristic flavor and aroma to the Parmigiano Reggiano cheese and provide knowledge about the quality and safety of the product. The lipolysis of milk fat gives rise to the free fatty acids that impart a preferably sharp flavor to the cheese. In addition to providing an important contribution to the cheese flavor, these volatile free fatty acids also serve as precursors of other significant aroma compounds. In particular methylketones, alcohols, aldehydes, and esters. There was a significant rise in the concentrations of fatty acids, aldehydes, alcohols, and esters with the cheese maturation. Esters in the cheese are mainly originated from the enzymatic or chemical reaction between the fatty acids and primary alcohols [6] and thus the ester production is usually governed by the alcohol concentration. Primary and secondary alcohols contributed with characteristic fruity and nutty notes to the cheese flavor. Aliphatic aldehydes content was also high in the volatile fraction of the Parmigiano Reggiano cheese. No considerable difference in the volatile profile was found between the samples produced in different geographical areas, although few compounds—namely, 2-hydroxy-3-pentanone, hexadecane, 2-decenal, and 1H-indene 1-methylene—were identified only in the cheese samples from the mountains. This reveals the high degree of uniformity in the manufacturing procedures of Parmigiano Reggiano cheese in the restricted production area. The compounds 1-heptene 5-methyl, butanal 3-methyl, and 2,2,4,4-tetramethyloctane were found only in the undegraded high-quality cheese samples, whereas ethanol 2-butoxy and furfural were present only in the degraded cheese samples. There was a clear distinction in the flavor profiles between the undegraded and the degraded cheese samples, in a satisfactory correlation between the S3 and SPME-GC-MS methods. Moreover, the SPME-GC-MS analysis indicated both the qualitative and quantitative differences between the headspace composition of different classes of cheese samples.

## 5. Conclusions

The study of the volatile organic compounds (VOCs) and quality evaluation of Italian Parmigiano Reggiano cheese by a combined use of the nanowire sensor device S3, SPME-GC-MS, and sensory analysis produced some useful and promising results that help in assessing the cheese quality and controlling fraud. Of note in this study is that about 150 aroma compounds associated with different chemical classes were identified and analyzed that helped to discriminate a degraded or an adulterated Parmigiano Reggiano cheese from a typical one. The multivariate statistical analysis of the quantitative results with PCA linking the S3 measurements to the SPME-GC-MS results and sensory description of the samples enabled the study of differences in the volatile composition between cheese samples with different ripening times and classes.

The S3 device applied in this experiment is an easy-to-use, rapid, low cost, portable, non-destructive, and low power consuming versatile instrument for on-line or at-line analysis and screening of the cheese quality, that can be utilized to confirm if a given sample is following the production standards in order to assess the quality control. The proposed approach presented in this work could be beneficial to different scientific areas of food, medicine, and environmental monitoring.

## Figures and Tables

**Table 1:** Characteristics of the analyzed cheese samples within the present study.

Sample	Ripening Period (in Months)	Organoleptic Quality	Altitude Zone	pH
1	12	Degraded	Mountain ^1^	5.44
2	13	Degraded	Flatland ^2^	5.34
3	12	Undegraded	Mountain	5.41
4	12	Undegraded	Flatland	5.32
5	12	Undegraded	Flatland	5.36
6	16	Undegraded	Flatland	5.37
7	13	Undegraded	Mountain	5.48
8	13	Undegraded	Flatland	5.45
9	36	Undegraded	Flatland	5.44
10	36	Degraded	Flatland	5.30
11	18	Degraded	Flatland	5.30
12	16	Degraded	Flatland	5.38
13	16	Degraded	Flatland	5.33
14	16	Degraded	Flatland	5.40
15	16	Degraded	Flatland	5.42
16	36	Undegraded	Flatland	5.29
17	18	Undegraded	Flatland	5.39
18	16	Undegraded	Flatland	5.40
19	12	Undegraded	Flatland	5.40
20	17	Undegraded	Flatland	5.37
21	36	Undegraded	Mountain	5.32
22	16	Undegraded	Flatland	5.36
23	18	Undegraded	Flatland	5.38
24	18	Undegraded	Flatland	5.31
25	12	Undegraded	Flatland	5.31

^1^ Mountain: 90–600 m; ^2^ Flatland: <90 m.

**Table 2:** Sensor array characteristics, composition, morphology, and operating temperature.

Sensor Type	Sensor Composition	Morphology	Operating Temperature (°C)
SnO_2_–MoO_3_	Blend of SnO_2_ and MoO_3_	RGTO	245
ZnO	ZnO	Nanowire	280
SnO_2_	SnO_2_	Nanowire	375
SnO_2_//Ag	SnO_2_ catalyzed with Ag nanoparticles	RGTO	400
ZnO	ZnO	Nanowire	500
SnO_2_//WO_3_	Blend of SnO_2_ and WO_3_	RGTO	500

**Table 3:** List of volatile organic compounds (VOCs) identified in Parmigiano Reggiano cheese by SPME-GC-MS analysis.

Compound	Retention Time (min)	Relative Abundance
**Alcohols**		
Ethanol	2.100	1,271,909
(R)-(−)-2-Pentanol	5.140	1,041,682
2-Pentanol	8.666	437,882
3-Buten-1-ol, 3-methyl-	14.758	79,385
2-Hexanol, 5-methyl-	17.856	322,043
(±)-5-Methyl-2-hexanol	17.866	518,829
1-Pentanol, 5-methoxy-	17.890	52,461
1-Hexanol	19.094	92,232
Ethanol, 2-butoxy-	20.814	7,805
7-Octen-2-ol, 2,6-dimethyl-	22.632	74,098
2-Propyl-1-pentanol	24.067	39,338
2-Nonanol	25.065	53,071
2,3-Butanediol	27.010	405,842
Cyclohexanol, 1-methyl-4-(1-methylethyl)-, *trans*-	27.722	105,611
Ethanol, 2-(2-ethoxyethoxy)-	28.209	31,675
2-Furanmethanol	29.655	136,185
Phenylethyl Alcohol	36.585	55,055
2-Butanol, 1-benzyloxy-3-methyl-	36.634	32,202
1-Dodecanol	38.108	125,734
n-Tridecan-1-ol	38.128	19,231
**Aldehydes**		
Butanal, 3-methyl-	2.760	53,795
Furfural	23.145	12,006
Benzaldehyde	24.230	206,435
Benzeneacetaldehyde	28.627	179,525
2-Decenal, (E)-	28.716	16,977
2-Propenal, 3-phenyl-	34.250	27,352
**Ketones**		
2-Hydroxy-3-pentanone	3.290	4595
2-Heptanone	11.283	6,431,421
Acetoin	16.108	197,040
2-Nonanone	18.291	3,015,823
8-Nonen-2-one	22.119	311,544
4′,6′-Dimethoxy-2′,3′-dimethylacetophenone	25.136	28,165
2-Undecanone	27.311	367,514
3-Buten-2-one, 4-phenyl-	37.516	22,744
2H-Pyran-2-one, tetrahydro-6-propyl-	43.545	66,023
2H-Pyran-2-one, tetrahydro-6-pentyl-	43.555	46,553
Ethanone, 1-(3,4-dimethylphenyl)-	44.520	19,259
**Esters**		
Butanoic acid, ethyl ester	5.054	8,522,322
1-Butanol, 3-methyl-, formate	12.956	71,080
Hexanoic acid, ethyl ester	13.897	13,149,658
Pentanoic acid, 4-methyl-, ethyl ester	13.940	6297
Heptanoic acid, ethyl ester	18.147	30,246
Octanoic acid, ethyl ester	20.505	2,238,440
Methyl 5-acetyl-2-methoxybenzoate	25.046	88,972
Ethanol, 2-nitro-, propionate (ester)	26.477	90,749
Pentanoic acid, heptyl ester	27.884	77,044
Decanoic acid, ethyl ester	28.673	5,507,764
Propanoic acid, 2-methyl-, ethyl ester	30.985	52,758
Propanoic acid, 2-methyl-, methyl ester	31.080	18,892
p-Chlorophenyl benzylcarbamate	31.435	20,258
Propanoic acid, 2-methyl-, propyl ester	36.069	7322
1,2-Benzenedicarboxylic acid, bis(2-methylpropyl) ester	53.361	819,636
**Acids**		
Propanedioic acid, dihydroxy-	3.020	238,653
Acetic acid	23.015	7,740,104
Propanoic acid	26.330	180,981
Butanoic acid	28.938	43,581,336
Pentanoic acid, 3-methyl-	30.092	287,245
Butanoic acid, 3-methyl-	30.429	453,424
Pentanoic acid	31.843	11,805,742
Propanedioic acid, propyl-	32.294	6,824,393
8-Chlorocapric acid	32.527	17,089
Hexanoic acid	35.248	53,899,542
Heptanoic acid	38.295	1,046,044
Octanoic acid	40.970	11,434,240
Nonanoic acid	43.615	226,077
n-Decanoic acid	46.123	1,912,506
9-Decenoic acid	47.788	65,217
Dodecanoic acid	52.601	51,535
2-(Heptyloxycarbonyl)benzoic acid	53.390	6489
Benzoic acid	53.717	49,625
**Hydrocarbons**		
1-Heptene, 5-methyl-	2.390	153,314
Decane, 2,2-dimethyl-	2.432	14,203,627
Propane, 2-(ethenyloxy)-	3.586	1,158,015
2,2,4,4-Tetramethyloctane	4.330	463,598
Ether, 2-ethylhexyl tert-butyl	5.783	101,048
D-Limonene	11.659	32,651
Hexadecane	27.475	10,458
Heptadecane, 2,6,10,15-tetramethyl-	30.522	32,718
Tridecane, 1-iodo-	30.526	36,630
Eicosane	30.538	80,505
1H-Indene, 1-methylene-	31.415	2155
**Maillard products**		
Pyrazine, 2,6-dimethyl-	17.539	50,361
2,3,5-Trimethyl-6-ethylpyrazine	24.335	32,674
**Miscellaneous**		
N-Hydroxymethyl-2-phenylacetamide	36.638	14,590
l-Gala-l-ido-octose	38.358	1145
3,4-Anhydro-d-galactosan	52.615	2442
2-Propanol, 1-chloro-, phosphate (3:1)	58.172	192,862
